# Exposure to Pornographic Videos and Its Effect on HIV-Related Sexual Risk Behaviours among Male Migrant Workers in Southern India

**DOI:** 10.1371/journal.pone.0113599

**Published:** 2014-11-25

**Authors:** Bidhubhusan Mahapatra, Niranjan Saggurti

**Affiliations:** 1 Internation Centre for Integrated Mountain Development, Kathmandu, Nepal; 2 Population Council, New Delhi, India; Centre for the AIDS Programme of Research in South Africa (CAPRISA), South Africa

## Abstract

**Objective:**

Research on pornography and its association with HIV-related sexual behaviours is limited in India. This study aims to examine the prevalence and correlates of viewing pornographic videos and examine its associations with HIV-related sexual risk behaviours among male migrant workers in India.

**Methods:**

Data were drawn from a cross-sectional survey conducted in 2007–08 across 21 districts in four states of India. Respondents included 11,219 male migrants aged 18 years or older, who had migrated to at least two places in the past two years for work. Bivariate and multivariate methods were used to examine the association between viewing pornography and HIV-related sexual risk behaviours.

**Results:**

Two-fifths (40%) of the migrants had viewed pornographic videos in one month prior to the survey. Migrants aged 25–29 years, literate, unmarried and away from native village for more than five years were more likely to view pornography than their counterparts. Migrants who viewed pornographic videos were more likely to engage in paid (Adjusted odds ratio [AOR]: 4.2, 95% confidence interval [CI]: 3.7–4.8) and unpaid sex (AOR: 4.2, 95% CI: 3.7–4.7), report inconsistent condom use in paid sex (AOR: 2.3, 95% CI: 1.7–3.0) and experience STI-like symptoms (AOR: 1.7, 95% CI: 1.5–1.8) than their counterparts.

**Conclusions:**

The findings regarding migrants' exposure to pornography and its linkage with high HIV risk behaviour suggest that the HIV prevention programmes for migrants need to be more innovative to communicate on the negative-effects of viewing pornography. More importantly, programmes need to find alternative ways to engage migrants in infotainment activities during their leisure time in an effort to reduce their exposure to pornographic videos as well as risky sexual behaviours.

## Introduction

Recent evidence from India and elsewhere suggests that male migrants are at an increased risk of HIV infection [1],[2]. Migrants' HIV risk behaviours such as sex with female sex workers (FSWs) and inconsistent condom use with these partners at destination places have been identified as the primary reason for the high HIV positivity rate among them [1],[3],[4]. Research from India has demonstrated that a considerable proportion (17%) of male migrant workers reported sex with FSWs, of whom 40% reported inconsistent condom use with these partners in the 12 months prior to the survey [5]. Previous research reports that migrants initiate and engage in risky sexual behaviours in the place of destination due to separation from their family and spouse for extended periods [6]–[8], isolation coupled with loneliness [7], socio-cultural norms and the anonymity of living in a city [6],[7], and the nature of work [7],[9].

Empirical research suggests that in conjunction with the above mentioned socio-cultural factors, there could be various other environmental and psycho-social factors that could influence individual attitudes and behaviour. Empirical research among adolescents and adults from the general population suggests that exposure to pornographic material influences an individual's sexual risk behaviour. A review of empirical research suggests that exposure to pornography influences sexual attitudes, moral values, and sexual activity individuals [10]. Another meta-analysis of studies on pornography indicates that exposure to pornography is one of the factors contributing to the development of sexually dysfunctional attitudes and behaviours and puts exposed individual at increased risk for committing sexual offenses, and experiencing problems in his/her intimate relationships [11]. In addition, prolonged exposure to pornography can lead to exaggerated perception of sexual activity including cynical attitudes about love and sexual pleasure [12]. Moreover, studies in developed countries have demonstrated that exposure to pornography leads to the practice of risky sexual behaviour including negative attitudes towards condom, multiple sex partners, inconsistent or no condom use and sexual violence [12]–[15]. Individuals who frequently consume pornography have sexual aggression levels four times higher than those who do not consume pornography frequently [16].

As male migrants are vulnerable to HIV risk [1],[3], the influence of exposure to pornographic materials on their sexual risk behaviour needs to be examined. Few studies in India have explored the extent of exposure to pornographic materials in the general population or in any sub-group of the population. A recent study among young males and females reports that about one-third of males (35%) and very few (3%) females viewed pornographic films; and about one-quarter (23%) of males and 5% of females had access to pornographic books and magazines [17]. Another study from Tamil Nadu, India demonstrated that exposure to materials is a significant predictor of experience of sexually transmitted infections (STIs) among migrant hotel workers [18]. Apart from these studies, there is no research that examines the relationship between exposure to pornography and HIV risk behaviours in the Indian context. Given this context, this study aims to study the prevalence and correlates of viewing pornographic videos and examine its association with HIV-related sexual risk behaviours among male migrant workers in India.

## Materials and Methods

### Data

Data for this study were drawn from a cross-sectional survey conducted in 2007–08 across 21 districts in four states of southern (Andhra Pradesh, Karnataka, Tamil Nadu) and western (Maharashtra) India, identified as high epidemic states by the Indian National AIDS Control Organisation prior to the year 2005 [19]. The data was collected by Population Council, Karnataka Health Promotion Trust and Annamalai University and can be downloaded freely from the Harvard dataverse network [20]. Study participants were recruited through a two-stage systematic sampling procedure. Geographical maps drawn for each district were used to list all migrant worker residential areas and worksites and to select cluster areas within these sites for recruitment. Clusters were created by combining smaller sites and dividing larger sites such that each cluster offered an area with approximately 5000 male migrant workers. Three clusters were then selected randomly from within each district, and migrant men within the chosen clusters were systematically sampled to obtain a minimum of 2500 participants per state.

A total of 11,635 eligible male migrant workers satisfied the inclusion criteria- age 18 years or older, having migrated to at least two places in the past two years for work- were selected for the cross-sectional survey. Of these, 145 (1.2%) men refused to be interviewed, and 271 (2.3%) men did not complete their interview and were thus excluded from the analyses, providing our final sample size of 11,219.

### Ethics statement

Data were obtained through face-to-face interviews conducted in private locations close to the respondent's residence or workplace. Verbal consent was obtained from all respondents before being interviewed. Verbal consent was sought for two reasons: first, large proportions of male migrants were expected to be illiterates and cannot read and sign the consent form; second, no personal identifier or biological data were collected. The interviewers read the complete script of the consent form in to the respondent and explained if there was any doubt about any aspects of survey. For each respondent, a separate consent form was used and those who consented to be interviewed were asked to give their thumb impression on the consent form. Ethical approval for the study was obtained from the institutional review boards of the Population Council and the University of Manitoba, Canada.

### Measures

#### Exposure to pornographic videos

Respondents who had viewed either adult movies with sex content or blue films (using compact discs or shown in video parlours) in the one month prior to the survey were categorised as having exposure to pornographic videos (coded as 1); else considered as no exposure to pornographic videos (coded as 0). In this study, this variable was first used as a dependant variable to understand the predictors of viewing pornographic videos and then as an independent variable to examine the effect of viewing pornographic videos on HIV-related sexual risk behaviours.

#### HIV-related sexual risk behaviours

HIV-related sexual risk behaviours in the study were measured using the following indicators: paid sex (sex with a sex worker) and unpaid sex (sex with a female other than spouse who is not a sex worker), engagement in anal/oral sex, inconsistent condom use, experience of STI-related symptoms and alcohol consumption prior to sex.

All respondents were asked a question with dichotomous response categories (no, yes) to examine whether they had sex with a sex worker in the 12 months prior to survey. A similar question was asked to examine if respondents had sex with a non-sex worker other than spouse (hereinafter referred to as non-sex worker) in the 12 months prior to survey to assess unpaid sex. Questions were also asked on the type of sex acts they engaged in the last time they had sex with these partners. Individuals who reported either engaging in anal or oral sex were considered to have engaged in anal/oral sex in the last sex. Inconsistent condom use was assessed separately for sex workers and non-sex workers. For each type of partners, respondents were asked about frequency of condom use (indicated by 1 =  every time, 2 =  almost every time, 3 = sometimes, 4 =  never) during sex in the past 12 months. Individuals who had “always” used condoms in the last 12 months were coded as 0 (consistent condom users) and the rest were coded as 1 (inconsistent condom users).

The survey also collected information on self-reported STI symptoms. Participants were defined as having STI-like symptoms if they reported any of the following in the past 12 months: genital ulcers; swelling in groin area; itching in genital area; or frequent painful urination. To examine alcohol consumption prior to sex, a question with dichotomous response categories was asked to those who ever had sex.

#### Socio-demographic characteristics

Data collected included the socio-demographic characteristics of migrants, including age, highest level of education completed, marital status including cohabitation status with spouse, income, ability to save money, living arrangement, and frequency of return to the native place (origin). These variables were used as independent variables while predicting the likelihood of exposure to pornographic videos and as covariates while predicting the risk associated with the exposure to pornographic videos for different HIV-related sexual risk behaviours.

### Statistical analyses

Univariate, bivariate and multivariate analyses were performed. Univariate analysis was used to describe the profile of the study population. Bivariate analysis was used to present the prevalence of exposure to pornographic materials by socio-demographic characteristics. A series of multiple logistic regression models were generated; first to examine the predictors of viewing pornographic videos and second to examine the effect of viewing pornographic videos on HIV-related sexual risk behaviours. In the first logistic regression analysis, viewing pornographic videos was considered as the dependent variable and socio-demographic characteristics of migrants were considered as independent variables. In the rest of the logistic regressions, viewing pornographic videos was the key independent variable whereas socio-demographic factors were used as covariates and indicators of HIV-related sexual risk behaviours were considered as dependent variables. Results were presented in the form of percentages, adjusted odds ratios (AOR) and their corresponding 95% confidence interval (CI). All the analyses were carried out using STATA version 12.1 (StataCorp., College Station, TX, USA).

## Results

Table 1 suggests that respondents were about 27 years old (standard deviation [SD]: 5.5 years), living away from their place of origin for work for a period of about six years (SD: 4.5 years) and a quarter (25%) had high-school (class 10^th^) or above education. About half (51%) were currently unmarried and one-quarter (25%) of them were married and staying with their spouse. About one-third (34%) reported staying away from home overnight for work.

**Table 1:** Socio-demographic and migration related characteristics of male migrant workers in India, 2008 (N = 11,219).

Background characteristics	%ge or mean (SD)^1^
	(N = 11,219)
**Age (in years)**	
<25	41.2
25–29	33.5
30+	25.4
Mean (SD)^1^	26.6 (5.5)
**Education**	
Illiterate	14.9
Primary school	14.5
Secondary school	45.5
High school and above	25.1
**Marital status**	
Married and staying with spouse	25.0
Married but not staying with spouse	23.9
Not currently married	51.1
**Monthly income (in INR** 2 **)**	
< = 3000	39.4
3001–4000	32.2
4001+	28.3
**Number of individuals staying with**	
<5	45.3
5+	54.7
**Duration of migration (in Years)**	
<3	20.0
3–5	44.4
6+	35.6
Mean (SD)^1^	5.7 (4.5)
**Able to save money**	
No	41.5
Yes	58.5
**Stay away from home overnight**	
No	66.3
Yes	33.7
**Frequency of return of native place**	
Once per year	12.2
Few times a year	24.2
Many times a year	12.6
No specific schedule	51.0
Viewed pornographic videos in the past one month	39.8

1 SD: Standard deviation

2 INR: Indian Rupees

Two-fifths (40%) of the migrants had viewed pornographic videos in the one month prior to the survey. Table 2 suggests that migrants aged 25–29 years were more likely to report viewing pornographic videos than migrants who were 30 years or older (44% vs. 36%, AOR: 1.3, 95% CI: 1.1–1.4). Currently unmarried migrants were three times more likely to have viewed pornographic videos as compared to those who were currently married and staying with spouse (46% vs. 32%, AOR: 2.5, 95% CI: 2.1–2.9). Viewing pornography was positively associated with education and duration of migration. Viewing pornographic videos was also positively associated with education, duration of migration, inability to save money and staying away from home overnight for work.

**Table 2:** Unadjusted percent and adjusted odds ratios predicting effect of different socio-demographic and migration related characteristics on exposure to pornographic videos among male migrant workers in India, 2008 (N = 11,219).

Background characteristics	Viewed pornographic videos %(N)	AOR (95% CI)^1^
**Age (in Years)**		
<25	39.4 (4618)	1.0 (0.9–1.2)
25–29	43.5 (3753)	1.3 (1.1–1.4)
30+	35.6 (2848)	Referent
**Education**		
Illiterate	29.3 (1670)	Referent
Primary school	40.4 (1631)	1.5 (1.3–1.8)
Secondary school	37.0 (5106)	1.2 (1.0–1.4)
High school and above	50.8 (2812)	1.9 (1.6–2.2)
**Marital status**		
Married and staying with spouse	31.6 (2806)	Referent
Married but not staying with spouse	36.2 (2685)	1.2 (1.0–1.4)
Not currently married	45.5 (5728)	2.5 (2.1–2.9)
**Monthly income (in INR)** 2		
< = 3000	35.9 (4425)	Referent
3001–4000	38.4 (3614)	0.9 (0.8–1.0)
4001+	46.9 (3180)	1.2 (1.1–1.3)
**Number of individuals staying with**		
<5	36.7 (5079)	Referent
5+	42.4 (6140)	1.1 (1.0–1.2)
**Duration of migration (in Years)**		
<3	30.3 (2244)	Referent
3–5	40.4 (4984)	1.5 (1.3–1.7)
6+	44.5 (3991)	2.8 (2.4–3.2)
**Able to save money**		
No	49.3 (4654)	1.7 (1.6–1.9)
Yes	33.1 (6565)	Referent
**Stay away from home overnight**		
No	36.1 (7442)	Referent
Yes	47.1 (3777)	1.4 (1.3–1.5)
**Frequency of return of native place**		
Once per year	14.7 (1192)	Referent
Few times a year	36.2 (2369)	3.1 (2.6–3.8)
Many times a year	55.8 (1232)	6.5 (5.3–8.0)
No specific schedule	41.6 (4998)	3.7 (3.1–4.4)

1 AOR: Adjusted Odds Ratio, CI: Confidence Interval

2 INR: Indian Rupees

The associations between viewing pornographic videos and HIV-related sexual risk behaviours are presented in Table 3. Compared to individuals who had not viewed pornographic videos, those who had viewed such materials were more likely to engage in paid (8% vs. 24%, AOR: 4.2, 95% CI: 3.7–4.8) and unpaid sex (10% vs. 29%, AOR: 4.2, 95% CI: 3.7–4.7), report inconsistent condom use in paid sex (26% vs. 43%, AOR: 2.3, 95% CI: 1.7–3.0). Migrants who viewed pornography were less likely to report inconsistent condom use in unpaid sex than those who did not view it (74% vs. 88%, AOR: 0.4, 95% CI: 0.3–0.5). Viewing pornography was also positively associated with engaging in anal/oral sex in paid (AOR: 1.6, 95% CI: 1.1–2.4) and unpaid sex (AOR: 2.8, 95% CI: 1.9–4.3), consumption of alcohol prior to sex (AOR: 3.4, 95% CI: 3.0–4.0) and experience of STI-like symptoms (AOR: 1.7, 95% CI: 1.5–1.8).

**Table 3:** Unadjusted percent and adjusted odds ratios predicting effect of viewing pornographic videos on HIV-related sexual risk behaviours among male migrant workers in India, 2008 (N = 11,219).

HIV-related sexual risk behaviour	Did not view pornographic videos	Viewed pornographic videos	AOR (95% CI)^1^
	% (N)	% (N)	
Alcohol consumption prior to sex^2^	10.0 (4922)	27.9 (3270)	3.4 (3.0–4.0)
Had paid sex in last 12 months	8.3 (6753)	24.4 (4466)	4.2 (3.7–4.8)
Inconsistent condom use in paid sex in last 12 months^3^	26.3 (559)	43.0 (1090)	2.3 (1.7–3.0)
Engaged in anal/oral sex in last paid sex^3^	8.1 (559)	13.7 (1090)	1.6 (1.1–2.4)
Had unpaid sex with female other than spouse in last 12 months	10.1 (6753)	29.4 (4466)	4.2 (3.7–4.7)
Inconsistent condom use in unpaid sex in last 12 months^4^	88.0 (685)	73.5 (1311)	0.4 (0.3–0.5)
Engaged in anal/oral sex in last unpaid sex^4^	5.8 (685)	17.7 (1311)	2.8 (1.9–4.3)
Had STI-like symptoms in last 12 months^5^	48.7 (6753)	62.2 (4466)	1.7 (1.5–1.8)

1 AOR: Adjusted Odds Ratio, CI: Confidence Interval

2 Among those who ever had sex (N = 8192)

3 Among those who had sex with sex workers in last 12 months (N = 1649)

4 Among those who had sex with non-sex workers in last 12 months (N = 1996)

5 STI: Sexually Transmitted Infections

## Discussion

The issue of pornography has received a great deal of attention among researchers over the years, both in developed and developing countries. In developed countries ample information is available on pornography and the pathways by which it affects sexual behaviour; however, such research is limited in India. To the best of our knowledge, this is the first study in its entirety to examine the prevalence of viewing pornography among male migrant workers in India. This study found that two-fifths of migrants had exposure to pornographic videos in the one month prior to survey, which is a little higher than one of the previous study among young males [17]. The study also found that viewing pornography was significantly associated young age (25–29) and longer duration of migration along with several other socio-demographic characteristics. The study also demonstrated that exposure to pornographic videos was associated with HIV risk behaviours among male migrants; those exposed to pornographic videos were more likely to engage in behaviours that may increase their risk of acquiring HIV than others.

The study observed that male migrants aged 25–29 years were more likely to view pornographic videos as compared to their younger and older counterparts. This is contrary to studies in developed countries, which suggest that younger males, particularly adolescents were more likely to be view pornography. There could have been a possible under-reporting among younger migrants as viewing pornography is not socially accepted in India. Future research is required to confirm this finding. Moreover, migrant who were unmarried were more likely to view pornographic videos as compared to married ones. Interestingly, more than 90% of unmarried migrants were aged below 30 years. The study also found that the duration of migration is positively associated with exposure to pornographic videos. This finding supports previous research on how long-duration migration keeps migrant men away from their families for long periods of time; and the living arrangements and peer dynamics in such instances make them engage in risky leisure activities such as gambling, visiting bars or pubs, visiting brothels and watching pornographic videos [1]–[3] [5]. Post-hoc analysis suggests that about two-fifths of male migrant watch porn during leisure time with friends. Migrants with longer duration of migration may be viewing these videos to fulfil their sexual desires and fantasies to some extent. The study also found that factors such as education, inability to save money, and frequent return to the native place were positively associated with viewing pornographic videos. The association between watching pornographic videos and the inability to save money may be interlinked. For instance, those migrants who watch pornographic videos are less likely to save money as they may be spending their income to watch videos and participate in subsequent risky behaviours including alcohol use and visiting sex workers. Similarly, those watching pornographic videos may be visiting their native place more frequently in order to satisfy their sexual desires (or) vice versa. Post-hoc analysis suggests that migrants who visits several times in a year to their native place were more than two times more likely to have sex with an unpaid partner in last 12 months as compared to those who went to their native place only once in a year (19% vs 10%),

The study findings shows that viewing pornographic videos can increase the likelihood of engaging in HIV risk behaviours among male migrants. Migrants who had viewed pornographic videos in the past one month were more likely to report paid sex (sex with a female sex worker) and unpaid sex (sex with a female who is not a sex worker and other than spouse) and reported practicing risk sexual practices such as anal and/or oral sex than others. Past research from developed countries has also suggested that exposure to pornography is associated with increased acceptance of premarital and extramarital sex including paid sex [21]–[25]. A recent research in United States among adults has also shown that individuals who had visited sexually explicit websites were twice more likely to have multiple sexual partners than their counterparts [21]. Another study which looked into different aspects of pornography exposure historically from 1973–2010 also found positive association with multiple sexual partners and paid sex [24]. In congruence with our study findings, previous research has also demonstrated that viewing pornography to be positively associated with the practice of oral sex and anal sex [13],[22],[26]. These findings indicate that migrants tend to learn risky sexual behaviour from pornographic videos and also develop an attitude favouring multiple sexual partners, paid sex and extra-and pre-marital sex.

The study finding revealed an interesting pattern in condom use behaviour. Inconsistent condom use by migrants is relatively higher in unpaid sexual relationships than in paid sex. Further, inconsistent condom use with female sex workers was higher among those who viewed pornography as compared to those who did not. However, a reverse relationship was observed with regard to inconsistent condom use in unpaid sex. The increasing proportion of migrants reporting inconsistent condom use with female sex workers reflects the influence of exposure to pornographic videos. In most of the pornographic videos, condoms are rarely used, which could have influenced the behaviour of individuals who are exposed to these [15],[27]. A relatively higher proportion of migrants viewing pornography using condoms consistently in unpaid sex (although overall condom use is very low) corroborates research from outside India [24]. This could be because they seem to be better informed about the debilitating effects of non-use of condom, especially, unwanted pregnancy [28]. Moreover, the unpaid sexual partners with whom migrants have had sex in our sample are generally females from the workplace, women in the neighbourhood in destination areas or the female friends or relatives in the native place, and such relationships are clandestine in nature [8] [28]–[30]. Given the nature of partners and the higher level of knowledge of condoms among those viewing pornography, the higher use of condoms could be attributed to fear of unwanted pregnancy in such relationships [8]. Post-hoc analysis among the subset of migrants who had sex with unpaid partners suggests that a higher proportion of those who viewed pornographic videos were older, unmarried and educated than those who did not view pornographic videos. Recent research from India also indicates that migrants, particularly unmarried, who have a higher likelihood of fear of pregnancy in such relationships may choose to use condom [28]. Also, given that those viewed pornography are better educated, it can be assumed that they would have better knowledge of contraception methods. Nevertheless, additional research is required to understand why those who are exposed to pornography are also the ones who are more likely to use condoms in unpaid sexual relationships.

This study also found that migrants who had viewed pornographic videos consumed alcohol prior to sex. Consumption of alcohol prior to sex is a well-recognised factor that can increase HIV risk multiple times. Empirical research has linked alcohol consumption prior to sex to increased HIV risk behaviour. Based on findings from this study, one can suggest that individuals may be adopting such behaviour due to their exposure to pornography. Pornographic videos, where alcohol is usually shown to be a stimulator before sex, could have guided a certain section of migrants to practice such behaviour in their sexual life as well. A significantly higher proportion of male migrants reported experience of STI-like symptoms in the 12 months prior to the survey, similar to findings from other studies examining the effect of pornography on STIs [15],[31],[32]. This is explained by the fact that many of the individuals who had viewed pornographic videos were not using condom consistently with sex workers. Research from India suggests that STI/HIV is highly prevalent among sex workers in India [33]. Therefore, these individuals may have acquired infection from these sex workers. The other reason for high STIs can be attributed to the type of sex act migrants practiced. A higher proportion of individuals who viewed pornographic videos also reported engagement in anal/oral sex with their sexual partners. Previous research has demonstrated that practice of such risky sexual acts can increase the risk of STI by multiple times [34]–[38].

Although the study findings offer new insights in a little-researched area in India, these findings should be viewed in light of certain limitations. First, data were collected in a cross-sectional survey and hence, while associations between exposure to pornographic videos and HIV-related sexual risk factors are evident, the cause-effect relationship between them is difficult to establish. Second, the indicators used in this study relied on self-reports and there could be a chance of social desirability bias in reporting. The potential for social desirability influences were minimised by assuring participant confidentiality and conducting interviews in private locations without any other person's presence. Significant rates of high risk sexual behaviour were reported by participants, which suggest that they were generally honest in their responses. Third, our measure of exposure to pornography was associated with increased sexual risk behaviour; however, more detailed measures assessing pornography exposure are needed to fully explain this line of research. Future research studies should examine other dimensions of exposure to pornography, such as time spent viewing pornography, frequency of exposure, reasons for exposure, context of use, and norms and attitudes towards gender and sex. Fourth, we measured exposure to pornography only if respondents had watched adult movies or blue films. However, there could be several other types of pornographic materials, such as magazines, internet and books, which were not considered in this study. Fifth, the survey did not collect information on sexual attitudes and beliefs and therefore, we could not examine how viewing pornography influences migrants' attitudes and beliefs. We recommend that future research should collect these information along with behaviour data to draw more meaningful conclusions. Finally, this study was conducted among male migrants and hence, the findings may not be generalised to the general population in India. More research is required among the general population to establish if the findings of this study holds true. Nevertheless, the study findings are important from the point of view of India's HIV prevention programme.

Despite these study limitations, the findings of this study have several important programmatic implications. Even though pornography is illegal in India, the study findings reveal that a significant proportion of migrants have access to pornographic movies, and those exposed to pornographic videos have higher sexual risk behaviours. These results suggest the need for designing specific communication messages that focus on the need for safe sex in all relationships even though they are not shown in pornographic videos. Communication materials should also include the messages on negative temptations that can come post-pornography viewing, and its linkages with risky behaviours including consumption of alcohol prior to sex, and visits to sex workers, and unsafe sex. It may also be helpful to create infotainment activities for migrants, so that in their leisure time, they can get themselves engaged in these activities and get educated as well as entertained. Moreover, HIV prevention programmes while discussing issues of pornography should prioritise migrants who are middle aged, educated, currently unmarried, and staying away from native place for longer duration.

In conclusion, this is the first study to provide evidence on the relationship between exposure to pornography and HIV-related sexual risk behaviours among male migrants in India. Two in five male migrants have viewed pornography. The study demonstrates a positive relationship between viewing pornography and extra- and pre-marital sex. Also, the study notes that inconsistent condom use with paid partners is higher among migrants viewing pornography than their counterparts. Findings of this study suggest that there is a need for HIV prevention education addressing issues related to the negative effects of viewing pornography.
